# Proton Dynamics in Palladium–Silver: An Inelastic Neutron Scattering Investigation

**DOI:** 10.3390/molecules25235587

**Published:** 2020-11-27

**Authors:** Daniele Colognesi, Franz Demmel, Alessandra Filabozzi, Antonino Pietropaolo, Alfonso Pozio, Giovanni Romanelli, Alessia Santucci, Silvano Tosti

**Affiliations:** 1 Consiglio Nazionale delle Ricerche, Istituto di Fisica Applicata “N. Carrara”, via Madonna del Piano 10, 50019 Sesto Fiorentino, Italy; d.colognesi@ifac.cnr.it; 2 Science and Technology Facilities Council, ISIS Facility, Harwell Oxford, Oxfordshire OX11 0QX, UK; franz.demmel@stfc.ac.uk (F.D.); giovanni.romanelli@stfc.ac.uk (G.R.); 3 Università degli Studi di Roma “Tor Vergata”, Dipartimento di Fisica, Via della Ricerca Scientifica 1, 00133 Rome, Italy; alessandra.filabozzi@roma2.infn.it; 4 ENEA, Dipartimento di Fusione e Tecnologie per la Sicurezza Nucleare, via E. Fermi 45, 00044 Frascati, Italy; alessia.santucci@enea.it (A.S.); silvano.tosti@enea.it (S.T.); 5 ENEA, Dipartimento Tecnologie Energetiche, Via Anguillarese 301, 00123 S. Maria di Galeria (Rome), Italy; alfonso.pozio@enea.it

**Keywords:** palladium/silver membranes, hydrogen diffusion, inelastic neutron scattering

## Abstract

Proton dynamics in Pd_77_Ag_23_ membranes is investigated by means of various neutron spectroscopic techniques, namely Quasi Elastic Neutron Scattering, Incoherent Inelastic Neutron Scattering, Neutron Transmission, and Deep Inelastic Neutron Scattering. Measurements carried out at the ISIS spallation neutron source using OSIRIS, MARI and VESUVIO spectrometers were performed at pressures of 1, 2, and 4 bar, and temperatures in the 330–673 K range. The energy interval spanned by the different instruments provides information on the proton dynamics in a time scale ranging from about 10^2^ to 10^−4^ ps. The main finding is that the macroscopic diffusion process is determined by microscopic jump diffusion. In addition, the vibrational density of states of the H atoms in the metal lattice has been determined for a number of H concentrations and temperatures. These measurements follow a series of neutron diffraction experiments performed on the same sample and thus provide a complementary information for a thorough description of structural and dynamical properties of H-loaded Pd-Ag membranes.

## 1. Introduction

The development of appropriate processes for hydrogen separation from gas mixtures is a topic of great interest for many industrial applications in which hydrogen is present in the by-products stream. The literature provides examples of hydrogen recovery from the combustion gaseous stream obtained in the production of carbon black [1], the off-gas stream of the manufacturing process of solar fuel cells [2], complex mixtures produced by the methane reforming [3], and many other sources in which hydrogen has to be opportunely recovered in view of its use as a clean energy vector [4,5,6]. Most common hydrogen recovery techniques are based on pressure swing adsorption (PSA), cryogenic distillation (CD), and membrane separation [7,8,9,10,11]. The use of a given process is essentially driven by the purity of the final product (hydrogen) to be achieved, the production scale, and the cost. While PSA operates in batch mode, CD and membrane separation are continuous processes. Both PSA and CD processes are commonly used in large-scale commercial plants and are characterized by intensive energy consumption. Moreover, in CD, the presence of impurities such as nitrogen, water vapor, or oxygen may cause freezing and plugging in the pipelines. On the contrary, membrane separation processes are very energy- and cost-efficient for small/medium units, simple in operation, and compact. Therefore, membrane-based systems are considered very promising especially in recovering ultra-pure hydrogen (i.e., with a purity grade >99.999 vol.%) [12,13]. In this regard, the need of ultra-pure hydrogen is required in several fields such as in fuel cell applications (particularly proton exchange membrane, PEM), in the manufacture of semiconductors and of high-purity chemicals as well as in experimental fusion reactors, such as ITER (International Thermonuclear Experimental Reactor) and DEMO (“DEMOnstrating fusion power reactor”), where ultra-pure hydrogen isotopes undergo fusion reactions to produce energy [14].

In this framework, hydrogen-selective membranes based on palladium are one of the reference technologies. In order to avoid the well-known membrane embrittlement, an alloy of Pd and Ag is usually made [15]. Indeed, a Pd membrane, when hydrogenated, becomes brittle due to the lattice expansion following a certain number of α↔β hydride transformations. On the contrary, in Pd-Ag alloys, the lattice has been already expanded by the effect of Ag substitutional atoms, so that it is less influenced by hydrogen uploading than in the pure Pd case [16]. Alloying with Ag also increases the mechanical properties of the metal and its hydrogen permeability [17]. Actually, both tensile strength and permeability show a maximum for a silver content in the range of 20–40 wt %, which exactly corresponds to commercial Pd-Ag alloys used for membrane applications. It is also worth noting that even the electrical resistivity of Pd-Ag alloys exhibits a maximum in the range of 20–40 wt % silver content. In addition, various papers [18,19,20,21] agree that the trend with the H/M ratio of the electrical resistivity in Pd_x_-Ag_y_ (with *y* in the 0.19–0.29 range) is described by an “S”-shaped curve. Such a trend might depend on the existence (or co-existence) of different hydride phases: α phase for low H/M, (α + β) phases for higher H/M, and β phase for the highest H/M values. Nevertheless, the presence of the different hydride phases does not fully explain the “S”-shaped behavior of the Pd-Ag resistivity. As reported in Refs. [18,19,20], at least in principle, the inversion of the electrical resistivity at about 413 K could be explained considering the presence of two different H occupation sites in the Pd-Ag lattice: (1) a low-temperature hydride form, where hydrogen is mainly located in the *fcc* tetrahedral sites, which is expected to have a high resistivity; (2) a high-temperature hydride form, where hydrogen is mainly located in the *fcc* octahedral sites, featuring a low resistivity. Thus, the first maximum of the resistivity appears around room temperature for hydrogenated Pd-Ag alloys. A definitive check of this behavior needs a microscopic investigation in all the temperature range of interest, in order to establish the existence of tetrahedral occupancy by hydrogen atoms, which is an essential requirement of the mentioned model. In addition, experimental studies were performed to investigate the structural modification of Pd-Ag alloy induced by electrolytic hydrogen absorption as well as the influence of surface activation on the hydrogen permeation properties [22].

Another important macroscopic process is the hydrogen permeation [23,24] through the membranes whose investigation might provide information on the macroscopic diffusion coefficient. In fact, in hydrogen separation applications, diffusivity should control hydrogen permeation through the dense metal membranes when operating at high pressure and/or with thick membranes.

Hydrogen diffusivity at the micro scale (i.e., inter-granular diffusion) may depend on the concentration and the position of hydrogen atoms in the lattice (i.e., on the hydride form).

While the microscopic properties of pure Pd have been deeply investigated through neutron techniques [25], extensive studies on Pd-Ag alloy are rather scanty [26].

However, it is surely worth mentioning the pioneering neutron spectroscopic works performed by Chowdhury and Ross [27] (on Pd_0.9_Ag_0.1_H_0.55_ and Pd_0.8_Ag_0.2_H_0.45_ at *T* = 80 and 295 K), by Chowdhury [28] (on Pd_0.9_Ag_0.1_H_0.1-0.15_ at 498 K < *T* < 623 K and on Pd_0.8_Ag_0.2_H_0.2-0.25_ at 428 K < *T* < 573 K), as well as the more recent paper by Fratzl and coworkers [29] (on Pd_0.9_Ag_0.1_D_0.61_ at *T* = 100 K) and by Kolesnikov and coworkers [30] (on PdAgH_0.50_ and PdAgH_0.86_ at *T* = 24 K). As we can see, considering both the Ag concentration and the temperature range, only Ref. [27] seems somehow relevant to the present study. However, given the limited energy transfer range and resolution of the spectrometer available for the mentioned measurements, we could exploit these data only in a qualitative way.

Recent experimental investigations have been performed using neutron diffraction on Pd-Ag-D systems at the ILL (Institut Laue Langevin) research reactor [27,31], determining the phase composition of the system at different levels of hydrogen content (i.e., at different pressures and temperatures) as well as the kinetics of deuteration by in situ neutron diffraction [28,32]. In the last two studies, the hydrogen occupancy in the *fcc* octahedral site in various *T* and *p* conditions was determined [31], and the presence of two phases was also investigated following the reaction progress with time [32], in turn pointing out that the α→β phase transition at room pressure occurs slightly above 423 K.

The present experimental study aims at complementing the structural information achieved in the aforementioned diffraction experiments by investigating hydrogen dynamical properties at different time and length scales.

When the issue to be addressed is the interaction of different isotopes (H, D, T) in a lattice, it is not possible, for example, to use spectroscopic techniques based on electron scattering [33], since the isotopic species are similar, but it is necessary to resort to those based on neutron scattering.

A series of neutron-based analyses of the H behavior in these membranes are presented, probing the single-particle dynamical regime from long time scales to short ones by means of different spectroscopic techniques, namely: Quasi-Elastic Neutron Scattering (QENS) [34], Incoherent Inelastic Neutron Scattering (IINS) [35], Neutron Transmission (NT), and Deep Inelastic Neutron Scattering (DINS) [36].

As a matter of fact, QENS probes hydrogen diffusion dynamics at the molecular scale and is able to differentiate free diffusion from confined dynamics; IINS provides information on the hydrogen vibration dynamics measuring the density of states, also probed by NT, while DINS probes the single atom ultra-fast dynamics and directly measures the single atom mean kinetic energy.

## 2. Data Analysis and Results

### 2.1. Incoherent Inelastic Neutron Scattering

At the end of the neutron scattering experiment on MARI (see subsect. 3.1 for the experimental details), raw time-of-flight data were normalized to the incoming neutron counts of the monitor, purged of contributions from noisy tubes, and corrected for detector efficiency. Subsequently, processed time-of-flight data were transformed into energy transfer spectra, removing the well-known kinematic factor (*E*_1_/*E*_0_)^1/2^ [36] and performing an appropriate rebinning. Finally, the usual vanadium spectra normalization was implemented, taking into account the minute angular effect due to the Debye–Waller factor of this metal.

The outputs of this part of the data analysis procedure were the so-called Σ(θ,ω) spectra, ranging in the interval −100 meV ≤ ħω ≤ 100 meV in steps of 0.25 meV and particularly suitable for the following operations:

(i). fast neutron background evaluation and removal;
(ii). container and Pd-Ag alloy scattering subtraction (after considering the neutron beam attenuation by H);
(iii). sample self-shielding and multiple scattering evaluation and correction.

Point (i) was operated automatically through the routines available on site, while the metal scattering subtraction was carefully performed via a fitting procedure, independently for each angular dataset, aiming at removing any trace of Bragg peaks from the elastic-line spectral zone. As for point (iii) concerning sample self-shielding and multiple scattering correction, we have used the analytical approaches suggested by Sears [37]. However, this method needed two important inputs, which are related to the proton vibrational dynamics of the measured samples, namely the total scattering cross-section of the hydride compound and an estimate of the scattering law to be folded with itself in order to generate the actual multiple scattering contributions. Both physical quantities have been derived via the well-known Gaussian approximation [36] making use of a fictitious, although quite realistic, H-projected vibrational density of states, *Z*_H_(ω), built using the literature information on similar compounds. As the experimental determination of *Z*_H_(ω) in the various measured samples is the actual purpose of the present experiment, a final check was done at the end of the data analysis: the fictitious H-projected vibrational density of states was replaced by the respective experimental one, but no significant differences were found either in self-shielding or in multiple scattering estimates.

At this stage, Σ(θ,ω) spectra were transformed into constant-*Q* data through standard binning routines. This transformation made possible the determination of a complete inelastic spectral array at *Q* values ranging in the interval 5.1 Å^−1^ ≤ *Q* ≤ 6.7 Å^−1^ in steps of 0.2 Å^−1^. As processed neutron spectroscopic data essentially contained only incoherent scattering from H, they could be dubbed as *self-scattering law* [36], *S*_s_(*Q*,*ω*).

**Figure 1:**
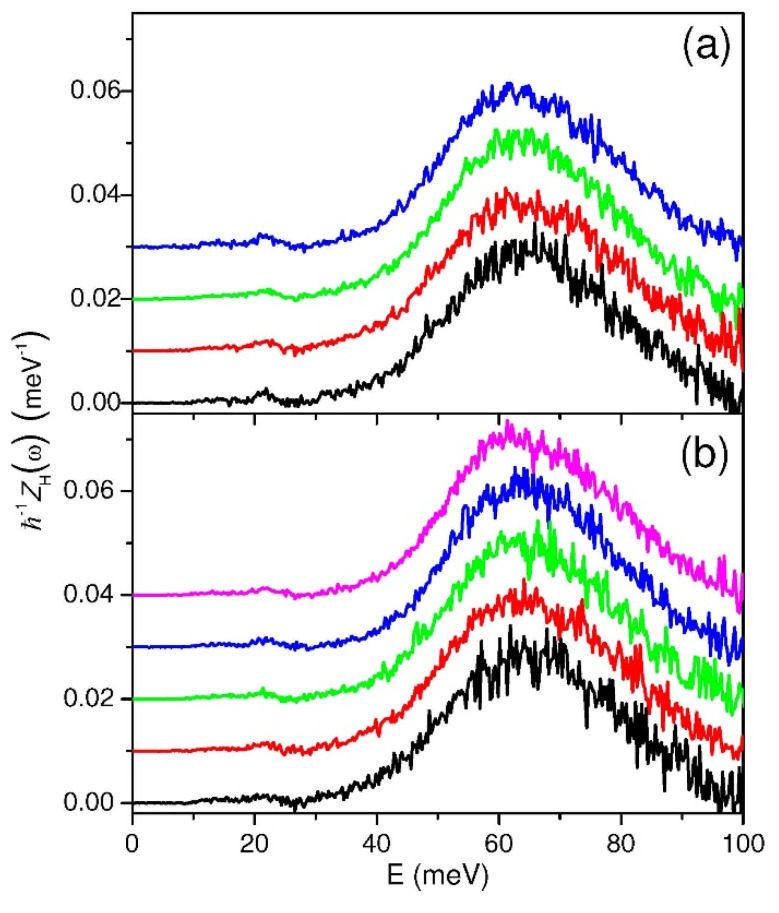
Experimental determinations of the H-projected vibrational densities of states, Z_H_(ω), for the samples reported in Table 1. Panel (**a**) contains data taken at *p* = 1 bar and *T* = 453 (black), 413 (red), 373 (green), and 333 K (blue). Panel (**b**) contains data taken at *p* = 2 bar and *T* = 453 (black), 413 (red), 373 (green), and 333 K (blue) plus at *p* = 4 bar, *T* = 333 K (magenta). Curves have been vertically shifted for graphic reasons.

In order to ease the rest of the data analysis, the so-called elastic lines, centered at ω = 0, were isolated and subtracted from all the *S*_s_(*Q*,*ω*) arrays. Subsequently, the new inelastic *S*_s_(*Q*,ω) arrays were transformed (following a well-known relationship [36]) into generalized H-projected vibrational densities of states, *G*_H_(*Q*,ω), which might retain a slight *Q*-dependence because of possible overtone/combination contaminations. However, by comparing all the *G*_H_(*Q*,ω) results at increasing *Q* values, it was verified that the overtone/combination contributions were negligible up to *Q* ≤ 6.7 Å^−1^ in all the measurements since the nine experimental estimates of *G*_H_(*Q*,ω), i.e., from *Q* = 5.1 Å^−1^ to *Q* = 6.7 Å^−1^, appeared almost identical within their statistical errors, exhibiting no actual *Q* dependence. At the end, by simply averaging all the *G*_H_(*Q*,ω) derived from the constant *Q* neutron spectra, the final experimental *Z*_H_(ω) was obtained for each sample (see Figure 1).

### 2.2. Quasi-Elastic Neutron Scattering

Data analysis included monitor normalization, detector efficiency correction, and conversion from time of flight to energy transfer. All data analysis steps have been performed using the MANTID toolkit [38]. Detectors have been grouped into 13 spectra. The energy transfer has been binned into constant 0.002 meV steps. As a result of the low scattering power, no multiple scattering corrections were necessary. Spectra have been analyzed with a fit model consisting of a single Lorentzian plus a delta function convoluted with the resolution function. In addition, a linear sloping background was fitted. The resolution function was derived from an unloaded Pd-Ag sample measurement.

**Figure 2:**
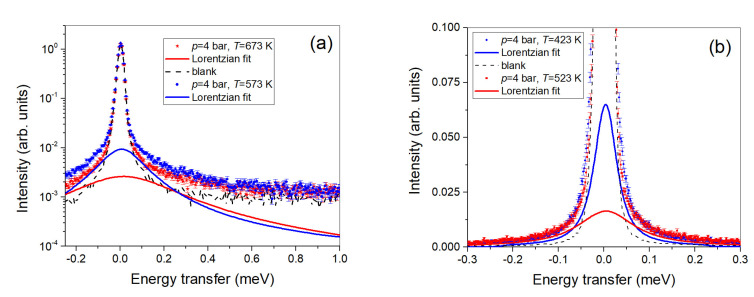
(**a**) spectra for the two highest temperatures at *Q* = 1.0 Å^−1^. The intensity scale is logarithmic to enhance the small quasi-elastic signal compared to the strong elastic scattering from the stainless-steel cell, which is included as a dashed line. The full lines show the fitted quasi-elastic contributions; (**b**) Spectra at *Q* = 1.0 Å^−1^ are shown on a linear scale at the two lowest temperatures. The signals of the blank and the fitted quasi-elastic contributions from hydrogen diffusion are also included.

Figure 2 shows examples of reduced data at *Q* = 1 Å^−1^. The left plot shows spectra obtained at the two highest temperatures (i.e., *T* = 673 and 523 K) with the asymmetric setting. The intensity is plotted on a logarithmic scale. The strong elastic scattering coming from the stainless-steel cell is to be compared to the blank spectrum, which is also included in the figure. Clearly, there is a weak quasi-elastic signal for the loaded sample, which broadens by increasing the temperature. The right plot shows the two lowest-temperature experimental spectra (i.e., at *T* = 423 and 523 K), including the unloaded sample (i.e., the “blank”) and the Lorentzian fits of the H quasi-elastic contributions.

**Figure 3:**
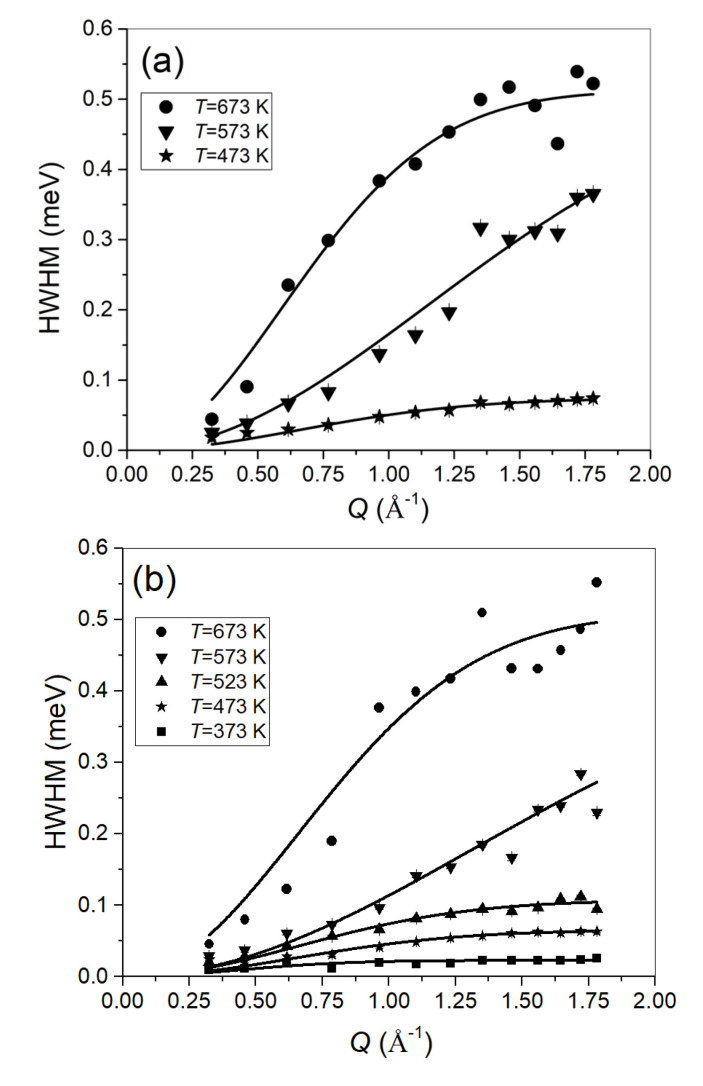
The half-width-at-half-maximum (HWHMs) for the 2 bar-loaded sample (**a**), and the 4 bar-loaded sample (**b**). Lines denote the fit with the mentioned Hall-Ross (HR) model.

From the Lorentzian fits, the *Q*-dependence of the half-width-at-half-maximum (HWHM) can be extracted. Figure 3 shows the evolution of the HWHM with *T* and *Q* for the 2 bar- and the 4 bar-loaded samples. PdH systems have successfully been fitted by applying the Chudley–Elliot (CE) diffusion model assuming jumps between octahedral sites [39,40,41]. The signature of the CE model is an oscillatory wavevector dependence of the width with the wavevector. Our data do not indicate any down-bending of the width with increasing wavevector within the covered wavevector range; hence, we have chosen the Hall–Ross (HR) model to describe our data. The HR model assumes a Gaussian distribution of jump lengths in contrast with the single one-jump length in the CE model. Both models converge to the Fickian diffusion law at small *Q*; however, the microscopic diffusion process might be described more realistically by a distribution of jump lengths in our case.

The HR diffusion model [42] (i.e., $\Gamma \left(Q\right)=\frac{h}{2\pi }\tau^{-1}(1-e^{-\frac{r_{0}^{2}Q^{2}}{2}}）$) [43,44] has been employed to fit the *Q* dependence of the jump diffusion process. Note that at *T* = 573 K, it seems that the *Q* dependence of the jump diffusion process changes in both measurements. Therefore, it does not appear to be related to a specific loading procedure variation during one of the measurement campaigns.

**Figure 4:**
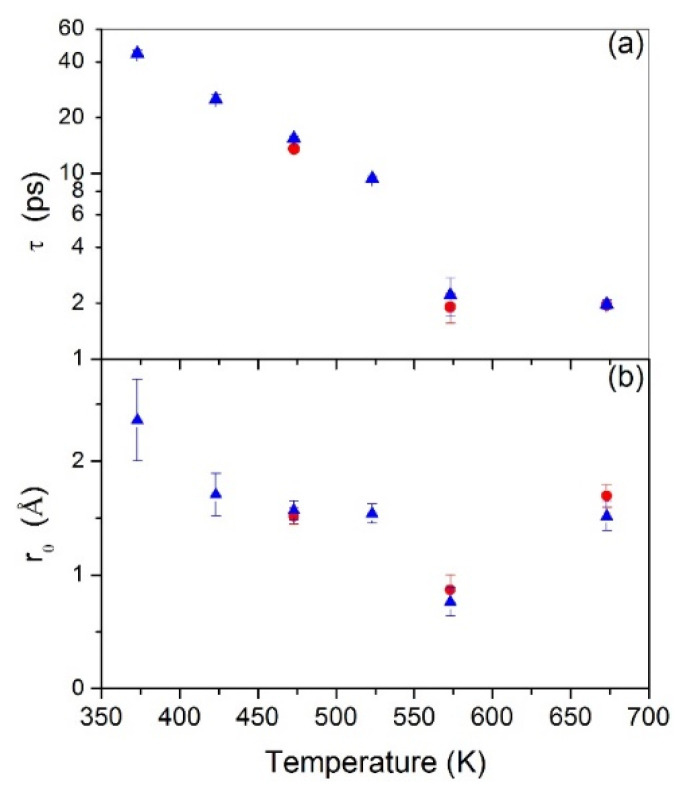
Residence times on a logarithmic scale (**a**) and jump length distribution (**b**), derived from the HR fit to the HWHM dependences at 2 bar (red full circles) and 4 bar (blue full triangles). For all loadings, the value at 573 K seems to deviate from the expected temperature trend.

**Figure 5:**
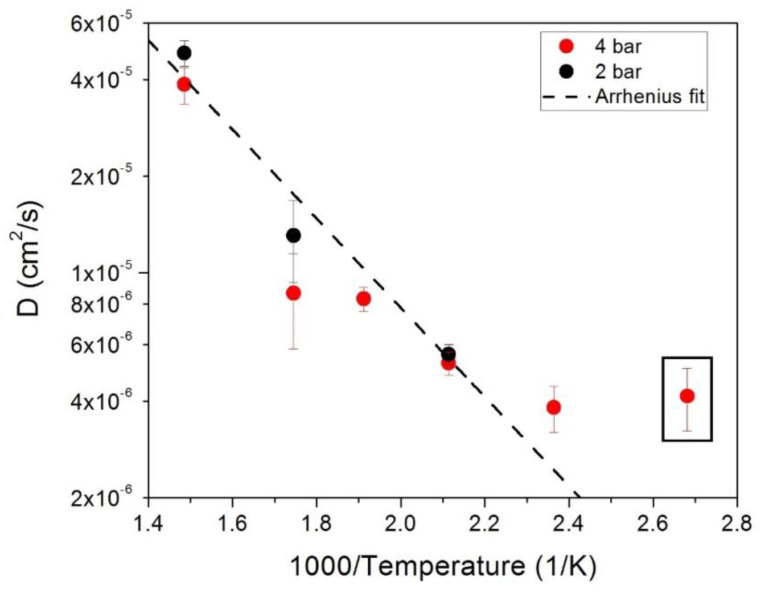
Calculated diffusion coefficients plotted against the inverse temperature in an Arrhenius plot. The line is a fit through all data points except the lowest temperature one (included in a black rectangle).

This change can also be seen by inspecting the obtained fit parameters in more detail. Figure 4 (left panel) shows the fitted residence times, *τ*, for both loading pressures on a logarithmic scale. The residence time at 573 K seems to deviate from the overall temperature trend. From the obtained *τ* and the jump length distribution *r*_0_ (reported in Figure 5), the diffusion coefficient *D* (reported in Figure 5) can be directly calculated according to $D=\frac{3\;r_{0}^{2}}{6\;\tau }$ [43].

In Figure 4, it can be noted that, for all loadings, the residence time has an Arrhenius-like behavior, except for the *T* = 573 K data points. As far as *r*_0_ is concerned, above *T* = 423 K (except for the *T* = 573 K data points), the average value is constant at about (1.6 ± 0.1) Å. From this value, a mean jump distance can be calculated according to $\langle r^{2}\rangle =3\;r_{0}^{2}$. We get as a mean jump length $\sqrt{\langle r^{2}\rangle }$ = (2.7 ± 0.2) Å, which demonstrates a good agreement with the octahedral jumps’ hypothesis instead of the tetrahedral jumps’ one [45]. Apparently, in Figure 5, the lowest temperature value at *T* = 373 K deviates from an Arrhenius-type behavior. This might be related to a change in the diffusion process at the lowest temperatures, or to the fact that the hydrogen dynamics cannot be fully resolved any more due to instrumental resolution limitations. A further study with a higher resolution spectrometer might be useful to clarify this point.

Except for the lowest temperature, where the fitted values are close to the energy resolution of the spectrometer, an Arrhenius-type process can describe the obtained diffusion coefficient. The line in the figure is a fit through all the points (except the lowest one).

From this fit, we can deduce an activation energy of about (120 ± 10) meV for the jump diffusion process. The same value of the activation energy has been related to the fast relaxation processes in PdH (see Ref. [45]).

At 573 K, the results from the fit apparently deviate from the other values and indicate a change in dynamics. Both measurements at 2 bar and 4 bar show a change in the diffusion process at 573 K through their evolution of the HWHM with *Q* (see Figure 3), which consequently leads to a change in the resulting jump lengths and diffusion coefficients. Since this change occurs in both independent measurements, we suggest that this is a genuine response from the sample and is not related to experimental inaccuracies.

**Figure 6:**
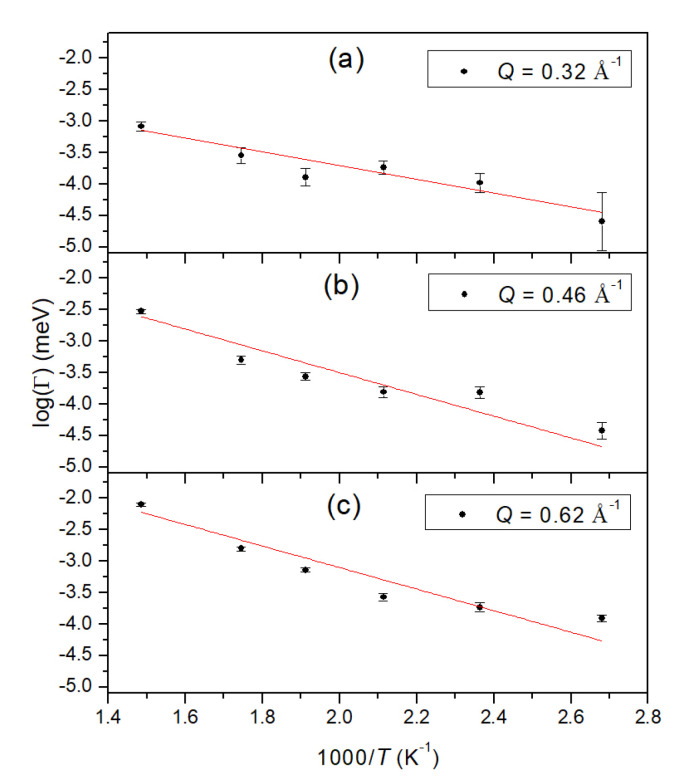
Measured full-width-at-half-maximum (black circles with error bars) plotted against the inverse temperature in an Arrhenius plot for the three smallest *Q* values. Red line is a fit through all data points. (**a**) data for *Q*= 0.32 Å^−1^; (**b**) data for *Q*=0.46 Å^−1^; (**c**) data for *Q*=0.62 Å^−1^.

To get more insight into the possible dynamics changes around 573 K, we plot in Figure 6 the width against the inverse temperature for the three smallest *Q* values. Included as a line is a fit for an Arrhenius process. There exists evidence that a fit with a single activation energy is not sufficient to describe the temperature dependence, in particular around 1.8 K^−1^, which corresponds to 573 K. It might be necessary to consider a change in the jump process around this temperature range. The jump length at this temperature (see Figure 4) is shorter than the other values.

This could be a hint that also jumps between tetrahedral positions play a role, where a smaller jump length of 1.95 Å is expected [40]. In a more recent study on the PdH system, it was claimed at around 573 K also tetrahedral positions are occupied [45]. To confirm or refute this idea, further experiments with a larger wavevector range might be in order, which might be a challenge due to the conflicting demands on larger wavevector range and high-energy resolution. From the other techniques presented here, no further evidence can be provided, because all the other measurements have been performed at a lower temperature.

### 2.3. Deep Inelastic Neutron Scattering

**Figure 7:**
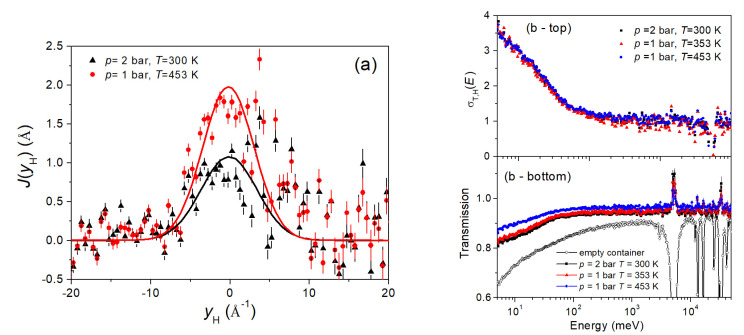
(color online): (**a**) The hydrogen neutron Compton profile at 2-bar loading and 300 K (black triangles), and at 1-bar loading and 453 K (red circles) in the West-scaling representation after background subtraction. Solid lines correspond to Gaussian fits with fixed widths using the Incoherent Inelastic Neutron Scattering (IINS) results (see main text for details); (**b**) neutron transmission (bottom right panel) and total cross-section (top right panel) for H in the previously mentioned conditions and at 1-bar loading and 353 K (red triangles), together with the corresponding signals for the empty Pd-Ag membrane (black line and empty circles).

As explained in detail below in the experimental subsect. 3.3, DINS spectra in time-of-flight appear as mass-separated peaks, also known as neutron Compton profiles (NCPs). The analysis of the recorded raw DINS time-of-flight data was performed using the mentioned MANTID software package, following the strategies described in Ref. [46]. Figure 7 shows an example of time-of-flight spectra for the forward-scattering VESUVIO detectors. In particular, the signals from Pd, Ag, and the container were removed by subtracting the empty-membrane spectra. One should also notice how the forward-scattering detectors on VESUVIO are gamma-sensitive (i.e., they count a neutron with a given final energy by detecting the prompt photon emitted by a gold foil analyzer), so measuring the actual scattering signal in addition to the photon background from the sample and the instrument environment. In the special case of this experiment, the gamma background during the DINS measurements was strongly enhanced by the presence of the Pd-Ag membranes, as both nuclei present strong prompt-gamma neutron-induced emissions. DINS data appear as a mass-resolved collection of peaks, each related to the single-particle dynamics of nuclei in the sample. Each peak has an intensity proportional to the number of nuclei of a given species in the sample multiplied by the associated total (bound) neutron cross section, whereas the width of the Gaussian-like peak is related to the mean kinetic energy of the nucleus of the considered species. The steps in the data analysis were the following: first, raw time-of-flight spectra for each forward-scattering detector were loaded into MANTID; then, corrected spectra were converted from time-of-flight to the hydrogen-related West-scaling variable, *y*_H_ [47]. In this domain, all the hydrogen peaks share the same position and display the same shape except for minor resolution-related and *Q*-related effects, and so these NCPs are readily interpreted as proportional to the one-dimensional proton momentum distribution. Figure 7 (left panel) shows the neutron Compton profiles for hydrogen in Pd-Ag at 2 bar-loading and 300 K, and at 1 bar-loading and 453 K, obtained by summing all spectra from the VESUVIO forward-scattering detectors. The multiple-scattering and Filter Cycling Technique (FCT) background corrections were neglected, considering the large sample-dependent gamma background. Therefore, in order to maximize the signal-to-background ratio, we have analyzed the sum of all hydrogen-containing spectra after subtracting the sum of all empty membrane spectra.

### 2.4. Energy-Resolved Neutron Transmission

In conjunction with the DINS measurements on VESUVIO, neutron transmission data were collected as a function of the incident neutron energy. The transmission spectrum of the container plus the empty membrane is shown in Figure 7 (right panels), and one can easily identify the neutron capture resonances from Pd and Ag for epithermal neutrons, as well as the component of the absorption cross-section that is inversely proportional to the neutron velocity. This spectrum was obtained using the so-called “empty instrument” (i.e., no sample in the beam) as a reference spectrum for normalization. The transmission spectra of hydrogenated samples at 300 K and 2-bar loading, at 353 K and 1-bar loading, and at 453 K and 1-bar loading are also shown, which are obtained using the empty membrane spectrum for normalization. In the top panel, the cross-sections of H contained in the Pd-Ag membranes are reported and normalized to the free scattering cross-section value (i.e., about 20.5 barn) for epithermal neutrons [36]. The normalization to the empty membranes, measured at 453 K, proved to be successful in all the energy ranges, except for the resonance peak centers, where pronounced temperature dependence is expected. Moreover, for neutron energies between 3 meV and 4 eV, the ratio of the measured hydrogen transmission spectra, after taking their value, proved to be constant, and it was fitted in order to estimate the relative amounts of hydrogen in the three experimental conditions investigated. One should also notice that neutron transmission experiments aiming at evaluating cross-sections are generally performed with flat-geometry samples. However, once a given geometry is fixed, its effect on the transmission spectrum can be defined as an almost constant factor that cancels out when spectral ratios are considered. Moreover, the saturated resonances of Pd and Ag at a few eV were used to characterize the measurement background, and its effect on the ratios is estimated as a systematic error of about 0.2%.

## 3. Experimental Procedure

The following presents the experimental procedure that has been applied in order to upload hydrogen into the Pd-Ag matrix at the different pressure (*p*) and temperature (*T*) values selected for measurements. The procedure can be summarized as follows:

1. Blank: sample cell containing unloaded Pd_0.77_Ag_0.23_ at the chosen temperature, *T*_0_;
2. Gas filling: keeping *T*_0_ constant, hydrogen gas (from CK Special Gases, 99.99% assay) is fed into the cell at the chosen pressure, *p*_0_;
3. Neutron measurements: along an isobaric line *p*_0_, data are recorded by decreasing the temperature, i.e., from the lowest to the highest H loading, in order to avoid hysteretic effects. Before starting the measurement at the chosen temperature, we wait for the system stabilization for about 1 h.
4. Hydrogen discharging: the sample is heated up to 600–700 K and H is pumped out under dynamic vacuum for about 1 h.

**Figure 8:**
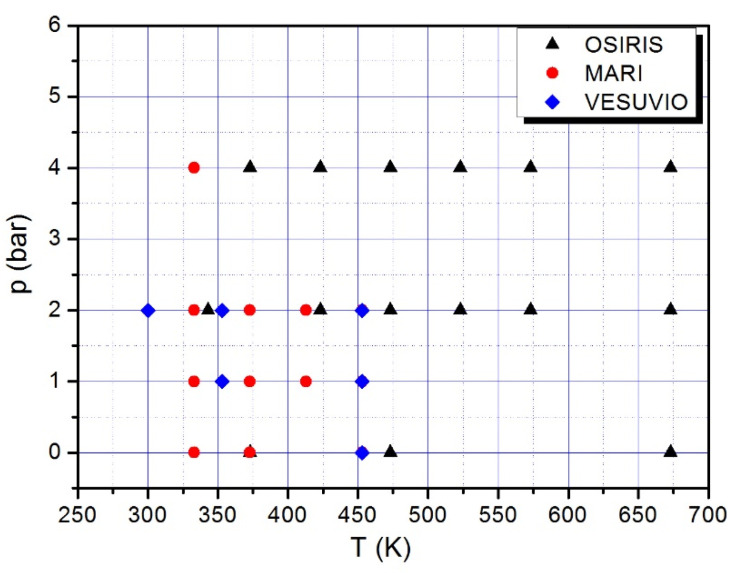
Pressures and temperatures selected for the different neutron scattering experiments. OSIRIS is for Quasi-Elastic Neutron Scattering (QENS) measurements, MARI for IINS, and VESUVIO for Neutron Transmission (NT) and Deep Inelastic Neutron Scattering (DINS).

The whole procedure was repeated for each temperature scan over the different isobaric lines (see Figure 8). The stability of the thermodynamic conditions during our measurements was acceptable, as temperature and pressure uncertainties were estimated to be around 1 K and 0.1 bar, respectively.

All containers used in the different experiments were chosen in order to have a cylindrical-slab geometry (i.e., an annulus) and consisted in two concentric cylinders giving rise to a 1.5-mm gap to be filled with the sample, showing an average diameter of 22.5 mm. As discussed below, the materials of the container walls were different for the various measurements performed. The mentioned “blank” sample was made of a set of Pd_0.77_Ag_0.23_ alloy slabs (from Goodfellow, 0.125 mm thick), which were squeezed inside the container.

### 3.1. Incoherent Inelastic Neutron Scattering on MARI

Incoherent inelastic neutron scattering measurements were performed on MARI [48], a spectrometer installed at the *ISIS Neutron and Muon Source* (Rutherford Appleton Laboratory, United Kingdom). MARI is a direct-geometry spectrometer based on the time-of-flight technique: the incoming neutron energy, *E*_0_, is selected by a Fermi chopper prior to the scattering event, while the final neutron energy, *E*_1_, is determined from the time of arrival of the neutrons, which are first scattered and then detected.

The angular range available on the instrument (i.e., 3.43° < θ < 134.13°) is almost continuously covered by ^3^He-gas detectors in steps of 0.43°. Using an appropriate value of the incident energy, *E*_0_ = 130.0 meV, we have been able to explore a wide zone of the (*Q*, ω) kinematic plane delimited by *Q* ≤ 14.5 Å^−1^ and ħω ≤ 100meV, with ħω being the energy transfer, and ħ*Q* being the momentum transfer modulus.

The MARI energy-transfer resolution, Δħω, is determined by the frequency and the type of the Fermi chopper employed in the measurements. In our case, a medium-low resolution option (i.e., the so-called “sloppy” chopper spinning at 550 Hz) was selected, resulting in values of Δħω at zero energy transfer (i.e., at the so-called “elastic line”) of about Δħω (0) = 3.4meV, which was experimentally determined via standard vanadium measurements.

As for the Δħω values at ω > 0, estimates obtained from routines available on the spectrometer were considered [49].

#### Experimental Setup

A comprehensive description of the measured samples (including temperature, pressure, hydrogen loading, and integrated proton current) can be found in Table 1. Temperature and pressure were monitored during the measurements, and hydrogen loading was calculated from these and the various Pd-Ag thermodynamic data available in the literature [21,31].

After performing the calibration measurement at room temperature making use of a standard vanadium rod, the sample cell (filled with an appropriate amount of Pd_0.77_Ag_0.23_ alloy, as explained later) was inserted into the instrument sample chamber equipped with a furnace. Then, we heated it up to the desired temperatures (i.e., *T* = 333,373, and 453 K), and we measured the neutron spectra of the so-called “blanks” (i.e., sample Nos. (1–3) as in Table 1). The mentioned “blank” sample of Pd_0.77_Ag_0.23_ alloy was amounting to a total mass of 62.1 g. Unlike other neutron spectrometers, the MARI detectors lie below the sample in a vertical scattering plane. Thus, the annulus axis was perpendicular to the neutron beam but horizontal rather than vertical as usual. The external diameter of the container, 25.0 mm, was much smaller than the vertical beam size (about 52 mm, including penumbra), while the container total length (i.e., 65.5 mm) was rather bigger than the horizontal beam size (still about 52 mm, including penumbra), so the cell was masked with boron nitride ceramics in order to exclude its bulky ends, which contained only screws, seals, and the stainless steel lid. In this way, the portion of the cell irradiated by neutrons was reduced to approximately 50 mm in length. Relying on the procedure described above neutron measurements of samples from No. (4) to No. (12) were performed as reported in Table 1.

### 3.2. Quasi-Elastic Neutron Scattering on OSIRIS

OSIRIS is an indirect time-of-flight near-backscattering spectrometer installed at the ISIS pulsed neutron source [50], combining two instruments in one: a high-flux indirect-geometry backscattering spectrometer and a long-wavelength diffractometer. For diffraction, the detector bank is composed of 962 ZnS pixels at 2θ ≈ 170°. For the spectrometer, the energy of the scattered neutrons is selected by means of Bragg scattering from a large-area pyrolytic graphite (PG) crystal-analyzer array, while neutrons are detected by 42 ^3^He gas detectors covering the angular range 11° < 2θ < 148°. The PG 002 configuration (final energy *E*_f_ = 1.845 meV) was used providing a *Q*-range of 0.18 ÷ 1.8 Å^−1^ and an energy transfer resolution of 25.4 μeV. Two different settings have been applied: a symmetric one with a dynamic range of ± 0.5 meV for the low temperatures and an asymmetric one (from −0.25 meV to 1.0 meV) for the two highest temperatures.

#### Experimental Setup

The measurement conditions for QENS and diffraction measurements (i.e., temperature, pressure, hydrogen loading, and integrated proton current) are reported in Table 2. Hydrogen uploading was calculated from data available in the literature [21,31].

A thin foil of unloaded Pd_0.77_Ag_0.23_ alloy was used in conjunction with an annular stainless-steel cell with an internal radius of 12 mm. The stainless-steel cell was necessary to achieve high temperatures together with the appropriate applied pressures. The mentioned single foil exhibited the following dimensions: 0.125 mm thick, about 70 mm wide and 40 mm high; it was inserted into the annulus, so that the total mass of the membrane in the beam was about 4.4 g. Even with the highest hydrogen loading, the total scattering power of the sample was only a few percent; therefore, a correction for multiple scattering events was not necessary. The cell was connected to a gas manifold in order to load the sample with hydrogen. Two unloaded-membrane (a.k.a. “blank”) measurements have been performed at *T* = 373 K and 673 K and served as an energy resolution function. Several temperatures have been achieved, ranging from 373 up to 673 K, implying different hydrogen loadings. Typical measurements lasted for about 10 h. The phase composition of the samples at the different *T* and *p* investigated is reported in dedicated papers [21,31,51].

### 3.3. Deep Inelastic Neutron Scattering and Neutron Transmission on VESUVIO

VESUVIO is an inverse-geometry electron-Volt neutron spectrometer [52] operating in the so-called Resonance Detector configuration [53] and making use of the foil cycling technique [54,55] for data recording. The spectrometer is placed along the line-of-sight of the room-temperature decoupled water moderator, which had been upgraded just before the measurement by removing one of the two poisoning Gd foils [56], providing an intense beam of epithermal neutrons (i.e., above the 410 meV cadmium cut-off energy), which are used to probe the short-time single-particle dynamics in condensed-matter systems [57]. Indeed, the range of energy and wave vector transfers achievable on VESUVIO in its peculiar inverse-geometry configuration are such that the impulse approximation is valid within a good degree of approximation. In this regime, the measured signal is separated in mass-resolved peaks, allowing for the measurement of the nuclear mean kinetic energy and, under suitable conditions, the determination of the effective local potential felt by the struck nucleus. The primary flight path from moderator to sample is about 11 m, while the detection system placed in the secondary flight path is composed of eight banks, each containing eight Yttrium-Aluminum Perovskite (YAP) scintillation detectors coupled to ^197^Au analyzer foils that select the neutron energy (after the scattering event) by means of the 4.906 eV nuclear resonance. Close to the sample tank, a movable frame allows for a second set of ^197^Au foils to be moved in and out of the trajectory between sample and detectors in order to implement the so-called foil cycling technique to reduce spurious background. Moreover, VESUVIO is equipped with incident and transmission ^6^Li-doped glass scintillator beam monitors, upstream and downstream of the sample position, respectively, which are used for total neutron cross-section measurements [58,59]. The white neutron beams available at VESUVIO, spanning from a fraction of meV to tens of keV, allow for broad-band and multi-purpose characterizations of materials, including elemental analysis.

#### Experimental Setup

Before the experiment, the membranes described above were heated in a furnace at 523 K in presence of ca. 1 bar of H_2_. Both the temperature of the sample and that of the furnace were monitored, and the latter during the baking process was kept at ca. 593 K. After a 21 h-baking, the sample volume was evacuated, still at high temperature, pumping for ca. 2 h; then, the pressure was stabilized at 10^−5^ mbar, the furnace was turned off, and a new sample cooling was started. This procedure was intended to avoid the presence of hydrogen from a previous *T*-*p* scan when starting a new one at different *T* and *p* conditions.

Neutron measurements were performed making use of a cryo-furnace, with the sample heated in the standard VESUVIO close-circuit refrigerator (CCR) with no exchange gas. In order to minimize the heat losses of the sample by irradiation and provide temperature stability, a 25-μm thick Al foil was wrapped around the cell. The temperature of the sample was measured by two sensors at the top and the bottom of the sample can (i.e., a stainless-steel annular container with a sample gap of 1 mm). Measurements were performed at 453 K in vacuum with 1 bar of H_2_ and with 2 bar of H_2_; at 353 K with 1 bar of H_2_; and at 300 K with 2 bar of H_2_, always following the procedure applied for the measurements on the other two neutron instruments.

## 4. Discussion

### 4.1. Incoherent Inelastic Neutron Scattering on MARI

Even a simple inspection of Figure 1 shows that all the measured samples exhibit qualitatively similar H-projected vibrational densities of states, as no visual changes can be detected either as a function of the temperature or of the pressure. The physical meaning of these experimental *Z*_H_(ω) estimates is quite straightforward: the weak low-energy region (say, ħω ≤ 25–27 meV) belongs to acoustic modes where protons move essentially in phase with the heavy atoms of the metal lattice, while the more intense part, stretching from 27 up to about 100 meV, includes the optic modes, where, on the contrary, proton vibrations are against the lattice atoms of the host alloy. A more quantitative assessment of the H-projected vibrational densities of states can be established by calculating the proton mean kinetic energy, <*E*_k_>_H_, from the Bose-corrected first moment of *Z*_H_(ω) [60,61]. The results reported in Table 3 show a clear positive trend with the temperature, which, as a matter of fact, can be easily explained as an effect of the Bose factor. A similar behavior is exhibited, as expected, by the H mean square displacement, <**u**^2^>_H_, still in Table 3, which can be directly calculated from *Z*_H_(ω) [36]. Both thermal variations are actually linear as largely expected, thus not providing new information on the system. Making use of standard formulas [36,60], it is possible to extract the purely quantum contributions to both <*E*_k_>_H_ and <**u**^2^>_H_, which are also known as *zero-point values* (labeled by the symbols <*E*_k,0_>_H_ and <**u**^2^_0_>_H_, respectively). In our case, their reasonable estimates, for example at *p* = 1 bar, are the following: <*E*_k,0_>_H_ = (48.3 ± 0.2) meV and <**u**^2^_0_>_H_ = (0.1034 ± 0.0005) Å^2^. These are interesting figures, since they can provide an experimental test of the hydrogen dynamics in the Pd-Ag system. As a matter of fact, the zero-point quantities comply with the Heisenberg uncertainty principle, which, in our notation reads: 2M_H_<*E*_k,0_>_H_ <**u**^2^_0_>_H_≥(9/4)ħ^2^, with the equality holding only in the case of a purely Einsteinian dynamics (i.e., a single harmonic oscillator). This represents the ideal case in which H is totally decoupled from the phonons of the metal lattice. Thus, any deviation from (9/4) ħ^2^ = 9.4054 meV Å^2^ amu can be interpreted as a mark of the H/Pd-Ag dynamical coupling. On the contrary, a large value of 2M_H_<*E*_k,0_>_H_ <**u**^2^_0_>_H_, namely (81/32) ħ^2^ = 10.5811 meV Å^2^ amu, stands for another idealized case, in which *Z*_H_(ω) is described by the Debye (i.e., parabolic) model, where the H vibration–lattice phonon coupling, although extremely simplified, is nevertheless very strong. In our case, one obtains 2M_H_<*E*_k,0_>_H_ <**u**^2^_0_>_H_ = (10.08 ± 0.06) meV Å^2^ amu, which lies almost in the middle of the two extreme cases, revealing a sizeable, although moderate, coupling between the H vibrational dynamics and the lattice phonons.

### 4.2. Quasi-Elastic Neutron Scattering on OSIRIS

The HWHMs reported in Figure 3 show a change in the *Q*-dependence of the jump diffusion process at *T* = 573 K both at *p* = 2 and 4-bar loading that reflects also in a possible deviation at *T* = 573 K of the *τ* values from the overall temperature trend (see Figure 4).

The values of the diffusion coefficient reported in Figure 5 are related to the jump motion of the hydrogen atoms and then describe the diffusion at the microscopic level. Differently, the hydrogen diffusion coefficients measured for Pd-Ag in experiments of gas permeation or electrochemical charging [51,62,63] are related to the macroscopic hydrogen mass transfer that takes into account a series of processes (lattice diffusion, grain boundary diffusion, etc.) in addition to the jump diffusion assessed above.

**Figure 9:**
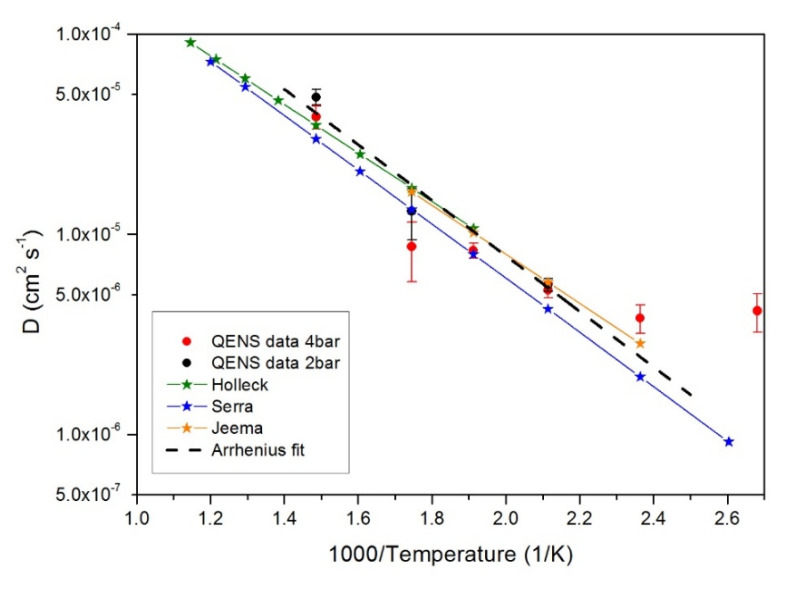
Macroscopic (full stars plus line) diffusion coefficients from Refs. [62] (green), [51] (blue), and [63] (orange) compared to QENS microscopic diffusion coefficients (full dots) at 2-bar loading (black) and 4-bar loading (red).

For a close comparison with the data available in the literature, we take into consideration data points from the present QENS measurements (reported in Figure 5) and the macroscopic diffusion coefficients derived from Refs. [51,62,63], reporting all these data in Figure 9.

A surprising, good agreement is found over the whole temperature range with the QENS data set. It is well-known that the hydrogen permeability into membranes (i.e., the macroscopic measurements shown in Figure 9) depends on the product of diffusivity (volume effect) and solubility (surface effect). QENS is sensible to the bulk diffusion process and not to surface or grain boundary-related effects, which also influence the macroscopic diffusion coefficients. Hence, over a wide temperature range, apparently the diffusion process is dominated by the bulk diffusion of hydrogen in Pd–Ag. It is to be noted that above 2.3 K^−1^ (i.e., *T* ≤ 423 K), QENS data deviate from the Arrhenius line and seem to lie above the expected values from the macroscopic measurements. We conclude that at lower temperature, the bulk diffusion process is no more the dominating process and surface effects might hinder the macroscopic hydrogen mobility through the membrane.

QENS is sensitive to the translational diffusion of H atoms in the matrix for the examined system. Through the momentum transfer, QENS monitors the microscopic jump process. In this context, the characteristics of the jump between sites (times and lengths) can be affected by the possible changes from a hydride phase to another. Both measurements for 2 bar and 4 bar indicate a change in the diffusion process at 573 K through their evolution of the HWHM with *Q* (see Figure 3), which consequently leads to a change in the resulting jump lengths and diffusion coefficients. Since this change occurs in both independent measurements, we suggest that this is a genuine response from the sample, which is not related to experimental inaccuracies. A further comment can be made considering the measurements performed at ILL on the structural properties of the systems [31,32]. As a matter of fact, the ND results highlighted that above 423–453 K, the α/β phase coexistence is no more evident. As shown in Figure 9, below 423 K, there is a possible deviation from an Arrhenius behavior. This aspect needs more experimental investigation by extending the measurements at lower temperatures.

### 4.3. Deep Inelastic Neutron Scattering and Neutron Transmission on VESUVIO

From the analysis of the DINS data, the measured mean kinetic energy at room temperature and 2-bar loading, and at 453 K and 1-bar loading, were found to be compatible with the results from the IINS measurements. In particular, the value of the H mean kinetic energy was kept fixed to the estimates obtained from IINS measurement (namely, to the value at 453 K and 1-bar loading, and to the average of the two measurements at room temperature, respectively). Subsequently, a fit making use of a Gaussian momentum distribution was performed, with the peak intensity, *I*(*T*,*p*), as the only free parameter.

The ratio of the two fitted intensities was *I*(300 K, 2 bar)/*I*(453 K, 1 bar) = 1.71 ± 0.12. The same quantity was independently evaluated via a numerical integration of the time-of-flight data, obtaining 1.56 ± 0.23. Moreover, estimates of the hydrogen uptake were also obtained from the neutron transmission spectra, not suffering for the enhanced gamma background. We found *I*(300 K, 2 bar)/*I*(453 K, 1 bar) = 1.58 ± 0.01, which was in remarkable agreement with the estimates from DINS, and *I*(300 K, 2 bar)/*I*(353 K, 1 bar) = 1.43 ± 0.01. We notice that the values from neutron transmission could be affected by a few-percent underestimation due to the cylindrical geometry of the container.

Furthermore, the energy-dependent hydrogen total cross-sections in the three mentioned conditions, dominated by the inelastic contribution, do not show a sizeable temperature dependence, mirroring the similarities observed in the IINS spectra, and a change in shape of the cross-section at about 60 meV is probably related to the intense hydrogen optic vibrations against the lattice reported in the spectra of Figure 1.

In view of future investigations of similar systems, we notice how DINS measurements were deemed to obtain two pieces of information. First, DINS is a mass-resolved technique whereby the amount of hydrogen atoms in a sample can be accurately determined [64]. Second, the magnitude of the zero-point nuclear energy can be assessed by direct measurement of the nuclear kinetic energy. The first is a valuable piece of information in the framework of the characterization of hydrogen absorption in bulk materials.

Moreover, the assessment of the magnitude of zero-point energies can help modeling the hydrogen dynamics, as shown by recent simulation studies for diffusion in alpha-iron [65]. We notice that the mean kinetic energy results from IINS rely on several assumptions, including a harmonic interaction potential, and they are generally used as a prediction only. While the application of DINS proved challenging in the present case, because of the many nuclear resonances of Pd and Ag, our results are discussed to provide a general protocol for use of inelastic neutrons scattering techniques to characterize hydrogen adsorption in metals. In particular, we could successfully map the intensity changes in different thermodynamic conditions, yet we had to fix the value of the kinetic energy to the IINS prediction. In the same framework, we show how energy-selective neutron transmission can be applied to confirm both the relative amount of hydrogen in a given sample and probe the main vibrational features.

## 5. Conclusions

The hydrogen dynamics in Pd-Ag membranes was studied at different time scales ranging from pico- to sub-femtoseconds using various spectroscopic techniques, namely Quasi-Elastic Neutron Scattering (QENS), Incoherent Inelastic Neutron Scattering (IINS), Neutron Transmission (NT), and Deep Inelastic Neutron Scattering (DINS).

From QENS data, it can be inferred that in the experimental conditions investigated, the diffusion process is determined by the microscopic jump diffusion mechanism, which resulted in a good agreement between QENS diffusion coefficients and *D*-values from macroscopic techniques. Except at the lowest temperature, bulk diffusion is the dominating process for the hydrogen mobility in Pd-Ag membranes. The details from the jump diffusion modeling indicate a change in the jump process around 573 K, which needs further investigation.

As far as IINS is concerned, spectral patterns of the H/Pd-Ag system, in the energy transfer range 0 < *E* < 100 meV, have been measured at various temperatures (i.e., 333–543 K) and hydrogen loadings (i.e., 1–4 bar).

These spectral features were found relatively insensitive to the thermodynamic and loading conditions, as also qualitatively confirmed by the measurement of the energy-dependent cross-section using neutron transmission. From the medium-momentum transfer region (i.e., 5.1 Å^−1^ < *Q* < 6.7 Å^−1^) of these IINS spectra, the H-projected vibrational densities of states are extracted and used to evaluate important physical quantities such as the H mean kinetic energy and mean square displacement.

In addition, it is important to stress that these H vibrational state distributions might be readily employed as a stringent validation tool for possible simulation work on the H/Pd-Ag system, since, as shown by the literature [24], their shape is extremely sensitive to the fine details of the interaction between hydrogen and the Pd-Ag metal lattice.

The ultra-fast dynamics investigated by means of DINS, although not performed in fully optimized conditions, provides a further consistency check of the experimental findings from IINS. Indeed, the energy related to the (optic) H vibrations is the main contribution to the overall single proton mean kinetic energy measured via DINS.

These studies follow a series of neutron diffraction measurements [31,32] performed on the same samples and thus provide complementary information for a thorough description of structural and dynamical properties of H-loaded Pd-Ag membranes that find applications in the fuel cycle of fusion experimental reactors.

The good agreement found between the H diffusivity in the Pd-Ag matrix measured at the microscale and the one reported in literature and measured at the macroscale reveals that inelastic neutron scattering is a valuable tool for measuring this parameter whose knowledge is fundamental for the materials used as hydrogen separation membranes.

## Figures and Tables

**Table 1:** Thermodynamic conditions of the samples measured on MARI and experimental results, including sample number, temperature *T*, pressure *p*, hydrogen uploading (expressed as ratio, *r,* between hydrogen atoms and metal atoms), integrated proton current I.C. (the average ISIS proton current being about 180 μA). Refs. [21,31] provide the relationship between thermodynamic conditions and H concentration.

No.	*T* (K)	*p* (bar)	*r* (%)	I.C. (μA h)
(1)	453	0.0	0.0	600.04
(2)	373	0.0	0.0	600.01
(3)	333	0.0	0.0	554.24
(4)	453	1.0	9.2 [21]	1112.95
(5)	413	1.0	16.1 [21]	1295.43
(6)	373	1.0	31 [21]	1802.51
(7)	333	1.0	34 [21]	1362.43
(8)	453	2.0	19.8 [21]	800.85
(9)	413	2.0	18.4 [21]	1442.39
(10)	373	2.0	35.0 [21]	852.24
(11)	333	2.0	41.8 [21]	750.39
(12)	333	4.0	39.8 [31]	1117.21

**Table 2:** Thermodynamic conditions of the samples measured by QENS on OSIRIS and related experimental results, including sample number, temperature *T*, pressure *p*, hydrogen uploading (expressed as ratio, *r*, between hydrogen atoms and metal atoms [21,31]), and integrated proton current I.C.

Sample	*T* (K)	*p* (bar)	*r* (%)	I.C. (μA h)
**S1**	473	0.0	-	1240
	573	2.0	3.3 [21]	1730
	523	2.0	7.4 [21]	1900
	473	2.0	18.9 [40]	750
	423	2.0	20.3 [21]	750
	343	2.0	37.1 [21]	1460
**S2**	673	0.0	-	1050
	673	2.0	0.4 [21]	1800
	573	2.0	3.3 [21]	1380
	473	2.0	18.9 [21]	1800
**S3**	673	0.0	-	720
	673	4.0	3.2 [40]	2100
	573	4.0	7.9 [40]	1620
	473	4.0	35.1 [40]	1690
	373	4.0	58.4 [40]	1500
**S4**	373	0.0		2490
	523	4.0	20.7 [40]	1500
	423	4.0	48.3 [40]	1620

**Table 3:** Thermodynamic conditions of the samples measured on MARI and experimental results for H mean kinetic energy, <E_k_>_H_, and H mean square displacement, <**u**^2^>_H_. Statistical errors are also reported.

*T* (K)	*p* (bar)	<E_k_>_H_ (meV)	<u^2^>_H_ (Å^2^)
453	1.0	72.1± 0.9	0.173 ± 0.003
413	1.0	67.8 ± 0.7	0.171 ± 0.002
373	1.0	63.6 ± 0.5	0.159 ± 0.001
333	1.0	60.4 ± 0.4	0.145 ± 0.001
453	2.0	72.0 ± 0.9	0.176 ± 0.003
413	2.0	68.0 ± 0.6	0.160 ± 0.002
373	2.0	60.5 ± 0.6	0.151 ± 0.002
333	2.0	60.6 ± 0.6	0.139 ± 0.001
333	4.0	60.7± 0.5	0.138 ± 0.001
